# Expression profile and bioinformatics analysis of circRNA and its associated ceRNA networks in longissimus dorsi from Lufeng cattle and Leiqiong cattle

**DOI:** 10.1186/s12864-023-09566-0

**Published:** 2023-08-29

**Authors:** Chuang Yang, Longfei Wu, Yongqing Guo, Yaokun Li, Ming Deng, Dewu Liu, Guangbin Liu, Baoli Sun

**Affiliations:** College of Animal Science, South China Agricultural University, No.483 Wushan Road, Guangzhou, 510642 China

**Keywords:** RNA sequencing, circRNA, ceRNA network, Beef, Longissimus dorsi, Myofibers, Meat quality

## Abstract

This paper aims to explore the role of circRNA expression profiles and circRNA-associated ceRNA networks in the regulation of myogenesis in the longissimus dorsi of cattle breeds surviving under subtropical conditions in southern China by RNA sequencing and bioinformatics analysis. It also aims to provide comprehensive understanding of the differences in muscle fibers in subtropical cattle breeds and to expand the knowledge of the molecular networks that regulate myogenesis. With regard to meat quality indicators, results showed that the longissimus dorsi of LQC had lower pH (*P* < 0.0001), lower redness (*P* < 0.01), lower shear force (*P* < 0.05), and higher brightness (*P* < 0.05) than the longissimus dorsi of LFC. With regard to muscle fiber characteristics, the longissimus dorsi of LQC had a smaller diameter (*P* < 0.0001) and higher density of muscle fibers (*P* < 0.05). The analysis results show that the function of many circRNA-targeted mRNAs was related to myogenesis and metabolic regulation. Furthermore, in the analysis of the function of circRNA source genes, we hypothesized that *btacirc_00497* and *btacirc_034497* may regulate the function and type of myofibrils by affecting the expression of MYH6, MYH7, and NEB through competitive linear splicing.

**Supplementary Information:**

The online version contains supplementary material available at 10.1186/s12864-023-09566-0.

## Introduction

According to the Domestic Animal Diversity Information System (FAO-DAD-IS), there are over 70 cattle breeds widely distributed in China, with 53 of them being indigenous [1, 2]. Native breeds are divided into three categories in accordance with their geographical distribution, including northern-distributed group, central-distributed group, and southern-distributed group, which are distributed in northern China, middle and lower reaches of the Yellow River, and southern China, respectively [3, 4]. Native Chinese cattle were once bred as draft animals, which has allowed them to adapt to the climate in which they grow and to be highly resistant to adversity [2]. Natural selection and environmental characteristics have shaped the genomes and expression profiles of cattle grown in each environment. Cattle that have been grown in southern China for a long time are adapted to hot and humid climates, and they have excellent meat quality and high disease resistance [5, 6].

Meat quality is an important element in the assessment of the meat value of livestock [7]. Beef myofibers can affect the traits to a certain extent, such as tenderness, color, and pH drop of beef [8]. Thus, among the factors that can affect meat quality, myofiber is the strongest [9, 10]. However, the growth and development of muscle fibers include ontogeny at different embryonic stages, as well as hypertrophy and transformation at postnatal stages [9, 11]. In addition, the process of myogenesis is a complex biological process regulated by multiple factors, such as myogenic regulatory factors [12], signaling pathways [13], noncoding RNAs (ncRNAs) [14], and genes. Among these factors, circular RNA (circRNA) is an ncRNA with a covalently closed continuous loop structure that can play a role in gene regulation by competing with linear splicing [15], and this factor plays an important regulatory role in myogenesis [16]. In addition, the function of circRNA as a sponge of miRNA may indirectly affect the translation of mRNA [17]. CircRNA can be found in bovine muscle, and it is involved in the regulation of myogenesis [18–20].

Leiqiong cattle (LQN) and Lufeng cattle (LFN) are native cattle breeds in South China, which are primarily distributed in Guangdong and Hainan Provinces. These two kinds of cattle live in a subtropical climate environment for a long time, and they are less affected by artificial introduction. In addition, the breeding scale is considerable. The genetic relationship between LQN and LFN cattle is close, but their physical appearance, such as is quite different [5]. In this study, we compared the circRNA transcripts in longissimus dorsi from two types of southern Chinese cattle, then constructed and analyzed the circRNA-associated ceRNA network. These results could provide comprehensive understanding of the differences in muscle fiber development in cattle grown under subtropical conditions and extend our understanding of the molecular networks that regulate beef meat quality.

## Results

### Characteristics in meat quality and myofber

The result related to meat quality were shown in Table 1. Compared potential of hydrogen (pH) of longissimus dorsi from two varieties of cattle, pH value of LQC was significantly lower than LFC (*P* < 0.0001). As for the muscle color, the muscle lightness (L*) of LFC was significantly lower than that of LQC (*P* < 0.05), and the muscle redness (a*) of LFC was significantly higher than that of LQC (*P* < 0.01). It was no significantly differences between LFC and LQC on yellowness (b*) (*P* > 0.05). In the assessment of tenderness, tangential stress of LQC is lower than LFC (*P* < 0.05), that may preliminarily indicate that beef from LQC is more tender than that from LFN.

**Table 1:** Longissimus dorsi muscle quality about Leiqiong cattle and Lufeng cattle

Items	Varieties of cattle		*P-value*
	LQC	LFC	
pH (24 h)	5.66 ± 0.025^b^	5.83 ± 0.032^a^	0.000
L*	49.01 ± 3.937^a^	40.01 ± 3.011^b^	0.011
a*	15.35 ± 1.356^b^	19.51 ± 1.234^a^	0.004
b*	12.14 ± 0.494	11.64 ± 1.466	0.543
Tangential stress (Pa)	39.43 ± 14.228^b^	65.26 ± 22.305^a^	0.021
Cooking loss (%)	0.357 ± 0.008	0.343 ± 0.038	0.515

**Fig. 1:**
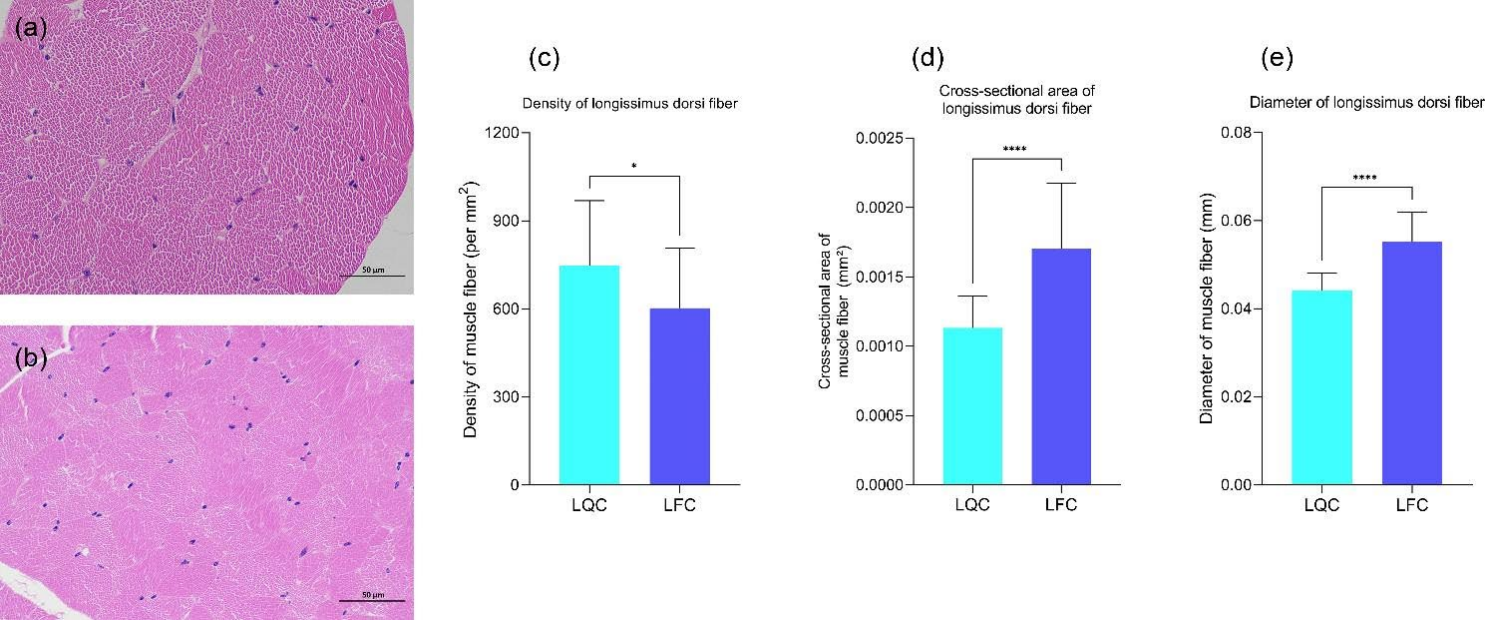
Longissimus dorsi myofibers difference between Leiqiong cattle and Lufeng cattle: (**a**) Section of longissimus dorsi muscle of Lufeng cattle; (**b**) Section of longissimus dorsi muscle of Leiqiong cattle; (**c**) Density of longissimus dorsi myofibers between Lufeng cattle and Leiqiong cattle; (**d**) Cross-sectional area of longissimus dorsi myofibers between Lufeng cattle and Leiqiong cattle; (**e**) Diameter of longissimus dorsi myofibers between Lufeng cattle and Leiqiong cattle. *P < 0.05, ****P < 0.0001

Compared the longissimus dorsi fibers from two kinds of cattle (Fig. 1), in the same level viewing area, myofibers quantity of LQC was significantly more than that of LFC (*P* < 0.05). For the area and diameter of longissimus dorsi fibers, LQC has smaller myofibers area and smaller diameter compared with LFC (*P* < 0.0001).

### miRNA and transcriptome expression analysis

**Fig. 2:**
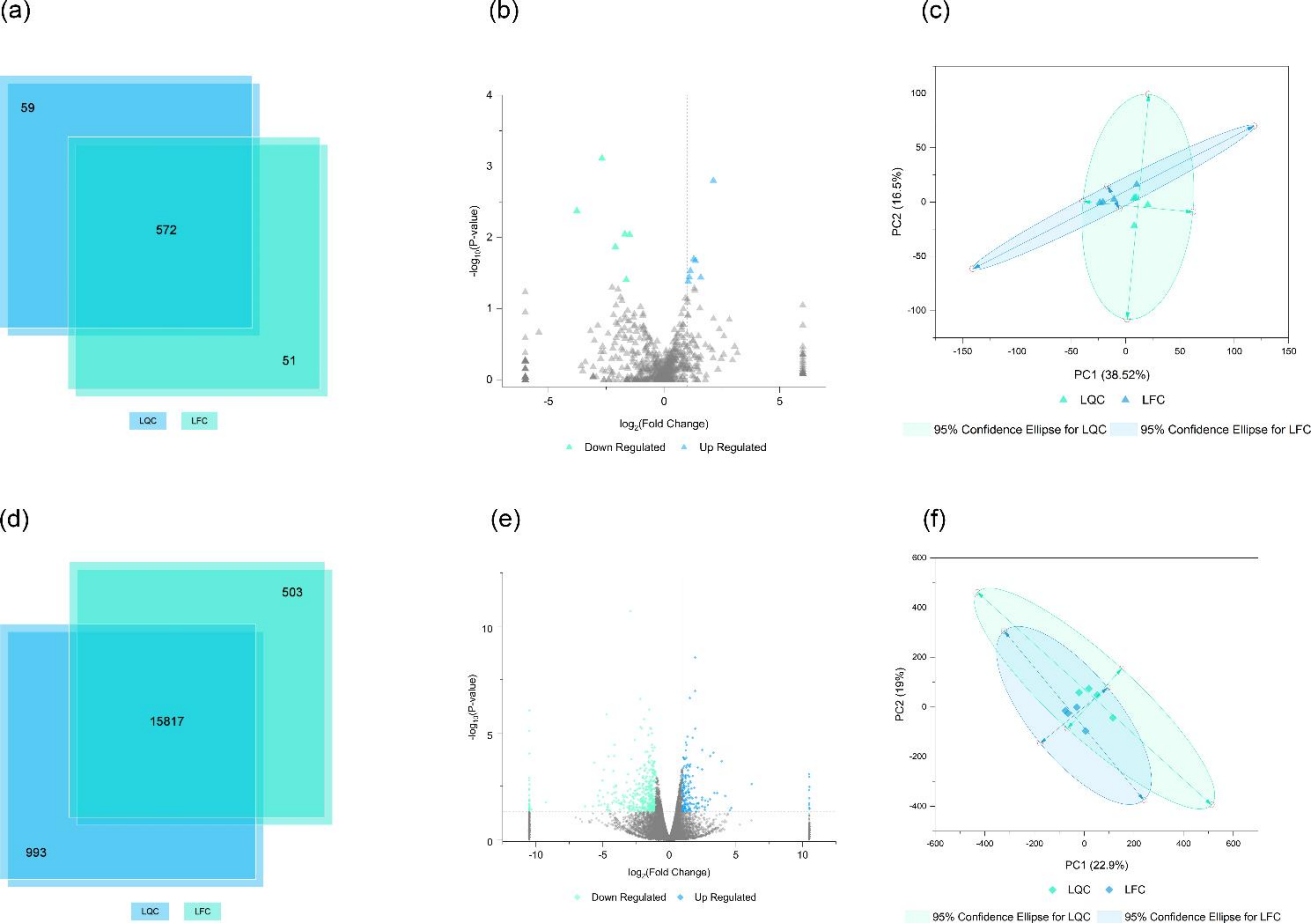
DE miRNAs and transcripts expression analyses of Longissimus dorsi from Leiqiong cattle and Lufeng cattle: (**a**) Veen diagram of the number of miRNAs in longissimus dorsi from two types of cattle;(**b**) Volcano plot for DE miRNAs in Longissimus dorsi between two types of cattle; (**c**) PCA plot for miRNAs expression in each sample; (**d**) Veen diagram of the number of transcripts in longissimus dorsi from two types of cattle; (**e**) Volcano plot for DE transcripts in Longissimus dorsi between two types of cattle; (**f**) PCA plot for transcripts expression in each sample

miRNA-seq generated 24,041,508 row reads from 8 samples, and after filtering, 23,312,472 clean reads were obtained, accounting for 97.00 ± 0.05% of the row reads. The reference genome, *Bos taurus*, gene data was downloaded in miRBase and analyzed using miRDeep2 software, 92.21 ± 1.88% of clean reads could be mapped to the reference genome, and a total of 493known miRNAs as well as 538 miRNA precursors were obtained. Predictive analysis of sequences not annotated to any information using mireap yielded 34.88 ± 8.22 new miRNAs (Table S1). Among all the annotated miRNAs, 59 and 51 miRNAs were unique to LQC and LFC, respectively, and 572 miRNAs were shared (Fig. 2(a)). Further analysis of miRNAs differentially expressed (DEseq, Expression difference fold: |log2FoldChange| > 1, Significance: *P-value* < 0.05) between LQC and LFC by the longissimus dorsi, with LQC as the control group, revealed 7 up-regulated and 6 down-regulated (Fig. 2(b)& Table S2).

For the RNA sequencing of mRNA and circRNA, a total of 8 samples from LQC and LFC yielded an average of 108,794,942 reads per sample. After quality control and trimming using Cutadapt, there are 94,857,498 per sample clean reads were yielded. TopHat2’s upgraded HISAT2 software (http://ccb.jhu.edu/software/hisat2/index.shtml) was used to map the reads, and 97.14%±0.28% of the clean reads can be mapped to the reference genome, Bos taurus (Table S1). A total of 17,313 transcripts were found in all samples detected, of which 16,810 transcripts were found in samples from LQC and 993 were unique; 16,320 were found in samples from LFC and 503 were unique; and 15,817 transcripts were shared by LQC and LFC (Fig. 2(d)). Differential analysis of gene expression was performed using DESeq (Expression difference fold: |log2FoldChange| > 1, Significance: *P-value* < 0.05), with LQC as the control group, 155 up-regulated and 444 down-regulated (Fig. 2(e)&Table S2). The PCA plot of miRNA and mRNA expression profile is shown in Fig. 2(c& f). GO (Gene Ontology) analysis revealed that these differentially expressed transcripts were significantly enriched (P < 0.05), and the vast majority (77.7%) of GO terms were Biological Process (Table S3).

### circRNA identification

**Fig. 3:**
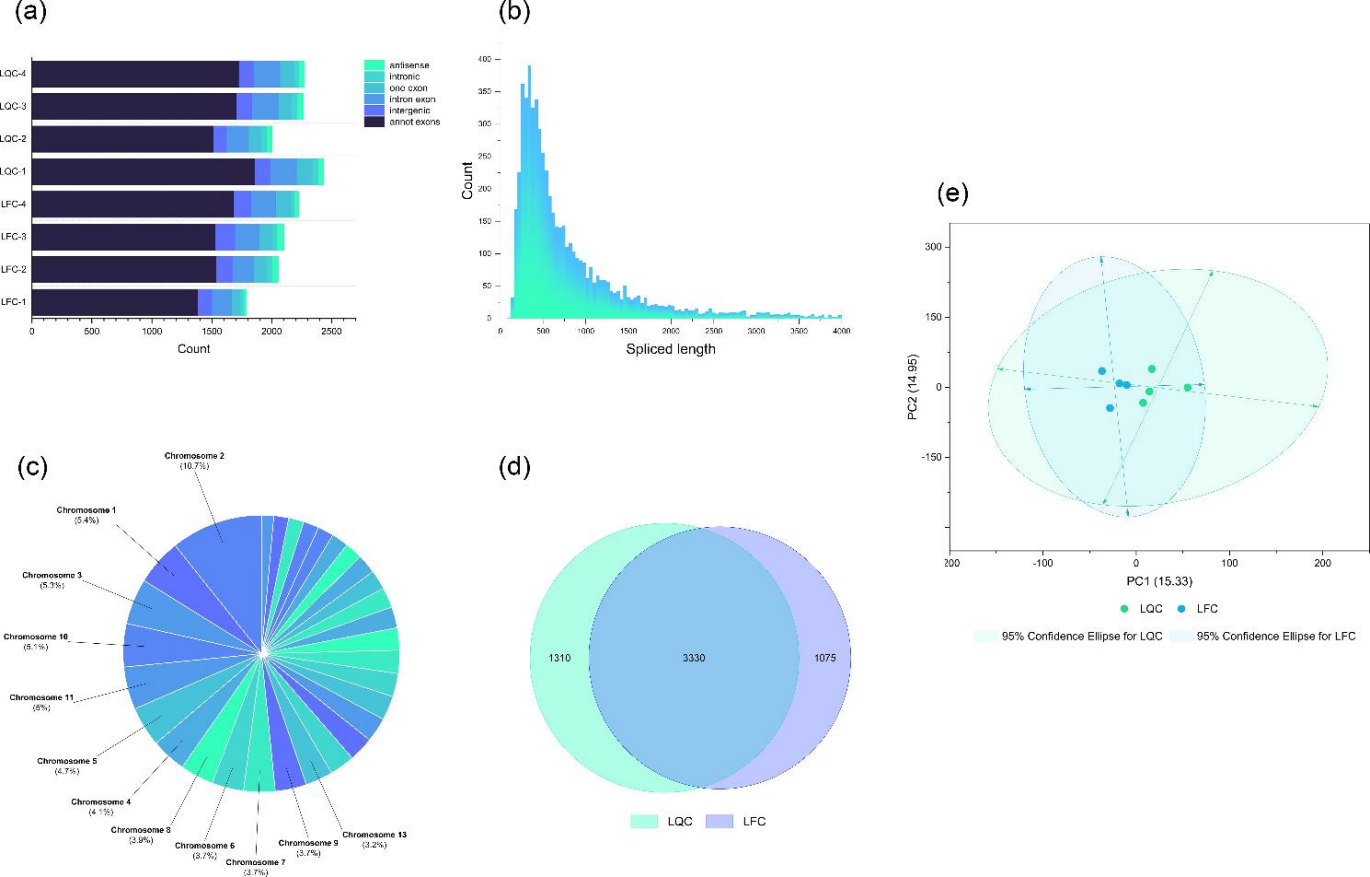
Expression profile about circRNA in Longissimus dorsi from Leiqiong cattle and Lufeng cattle: (**a**) The types of sourcegenes of circRNA that identified in each sample; (**b**) The spliced length of circRNA identified; (**c**) The distribution on chromosomes of sourcegenes of circRNA identified; (**d**) Veen diagram of the number of circRNA in longissimus dorsi from two types of cattle; (**e**) PCA plot for circRNA expression in each sample

After alignment with the reference genome, the unmatched Reads double-end 20 bp from the HISAT2 alignment results were intercepted as Anchors sequences and re-matched to the genome using Bowtie2 to detection of circRNA. 4,660,322 of these sequences were re-matched to the genome, representing 85.80 ± 3.50% of all Anchors sequences. Using find_circ to identify and annotate circRNAs, a total of 5,715 annotated entries were obtained (Table S4). Expression profile about circRNAs in LQC and LFC was showed in Fig. 3. The sourcegenes of identified circRNA vast majority were annotated exons (annot exons), and the spliced length of most identified circRNAs were concentrated about 500 bp (Fig. 3(a& b)). In the other hand, identified circRNAs were abundant on chromosome 1, 2, 3, 10 and 11. Compared with each other, there were 1,310 unique expressed circRNAs in LQC and 1,075 in LFC, 3,330 circRNAs were shared (Fig. 3(c& d)). PCA plot (Fig. 3(e)) showed that obviously separation exist between the samples of LQC and the samples of LFC, indicating that there was a certain difference in expression of circRNAs between the two types of cattle.

**Fig. 4:**
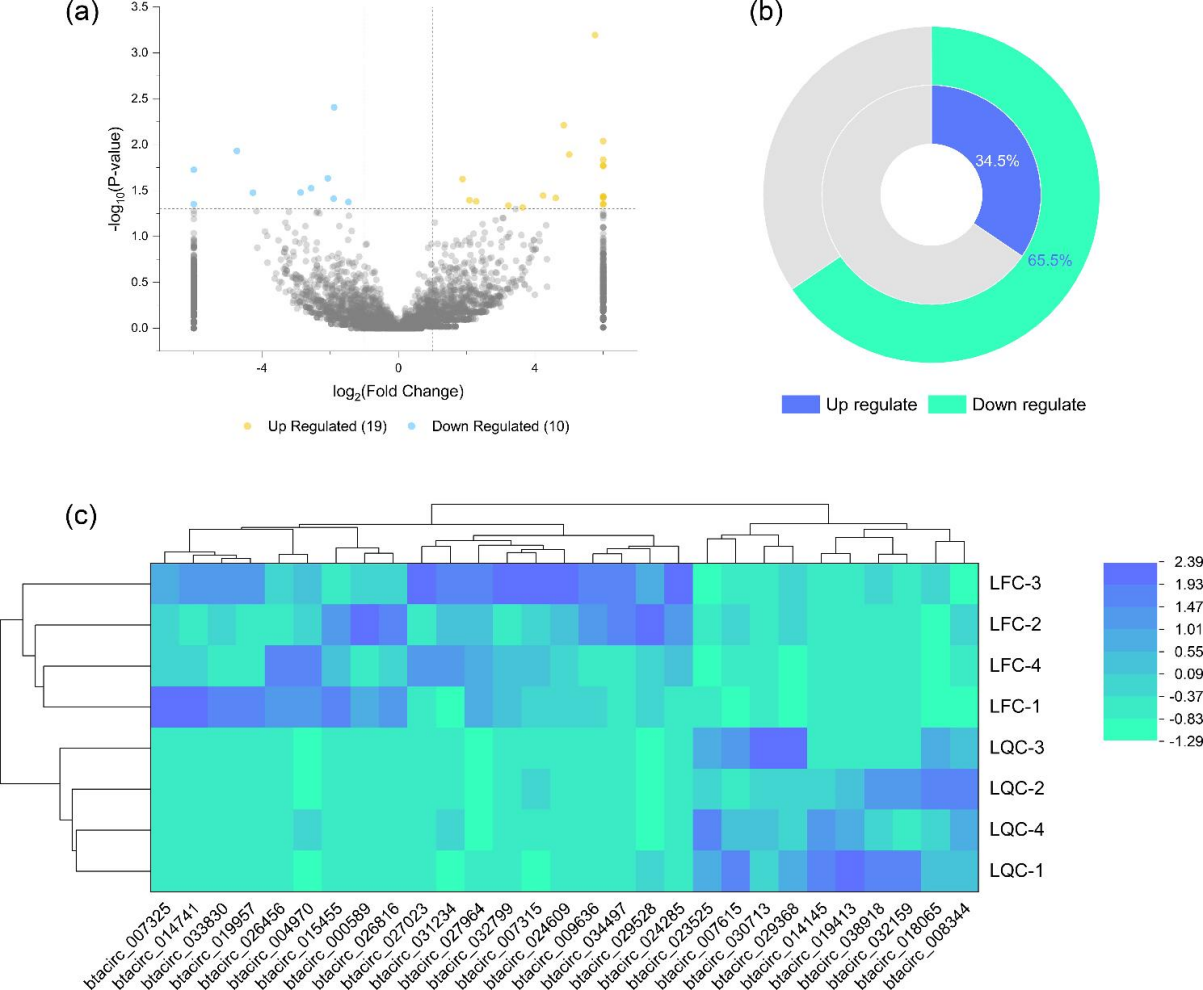
DE circRNAs between Leiqiong cattle and Lufeng cattle: (**a**) Volcano plot for DE circRNA between two types of cattle; (**b**) Ring diagram about the percentage of DE circRNAs between two types of cattle; (**c**) Heatmap for DE circRNA between two types of cattle

Furthermore, differential expressed circRNAs (DE circRNAs) between LQC and LFC were showed in Fig. 4& Table S2 (DEseq, Expression difference fold: |log2FoldChange| > 1, Significance: *P-value* < 0.05). Compared to LQC, 19 circRNAs were up regulated and 10 were down regulated in LFC (Fig. 4(a& c)), and accounts for 65.5% and 34.5% of all DE circRNAs (Fig. 4(b)), respectively.

### Construction of ceRNA-networks and Enrichment analysis

**Fig. 5:**
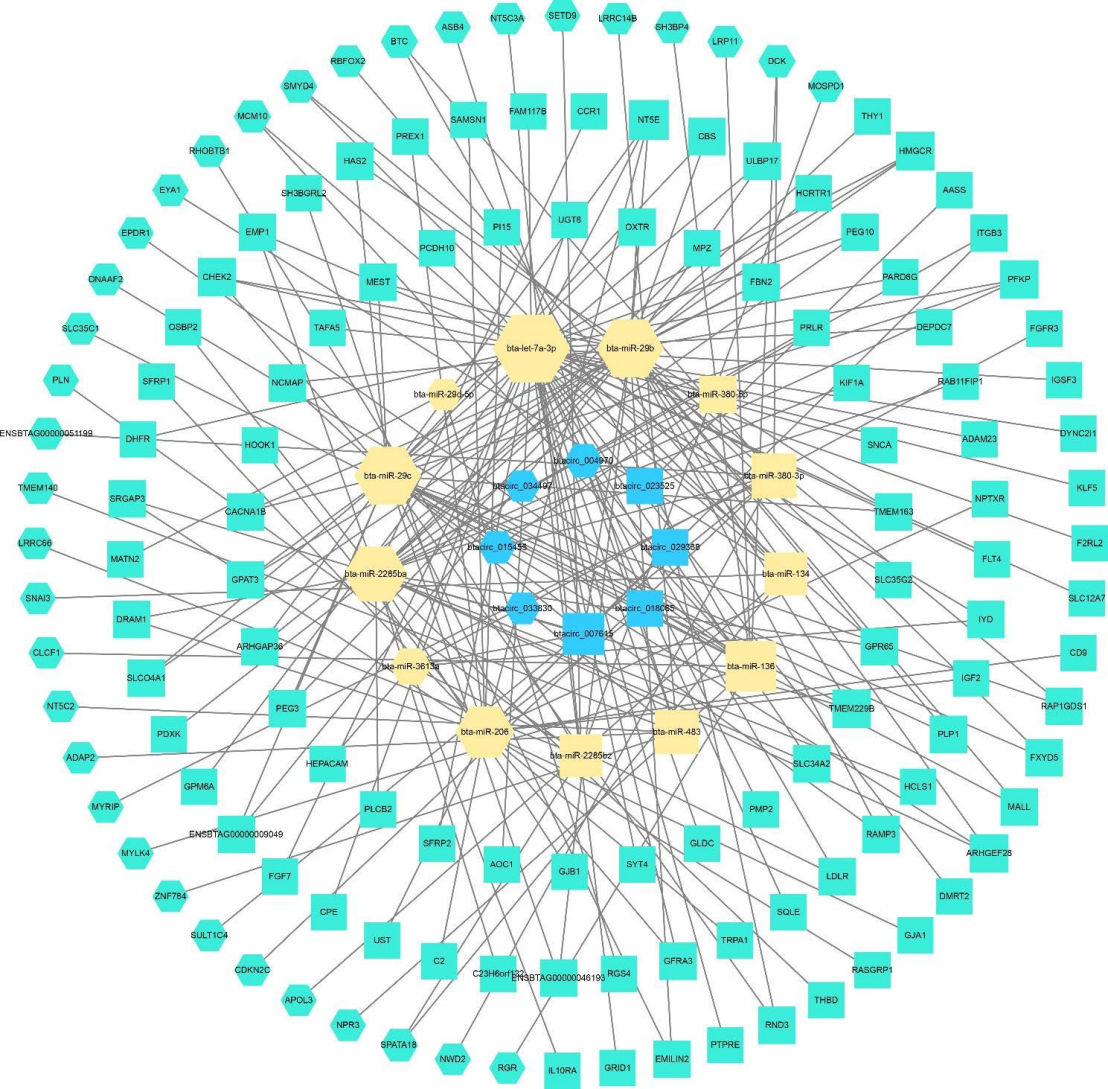
Identification of circRNA-associated ceRNA networks

After screening the DE circRNAs, psRobot and miRanda databases were used to predict targeted miRNA. As the co-expression networks in Fig. 5 and Table S5 shown, 13 miRNA and 135 mRNAs were associated with 8 circRNAs. Above them, there were 4 up-regulated and 4 down-regulated circRNAs, and influenced by them, 7 miRNAs and 35 mRNAs were up-regulated, and 6 miRNAs and 100 mRNAs were down-regulated.

**Fig. 6:**
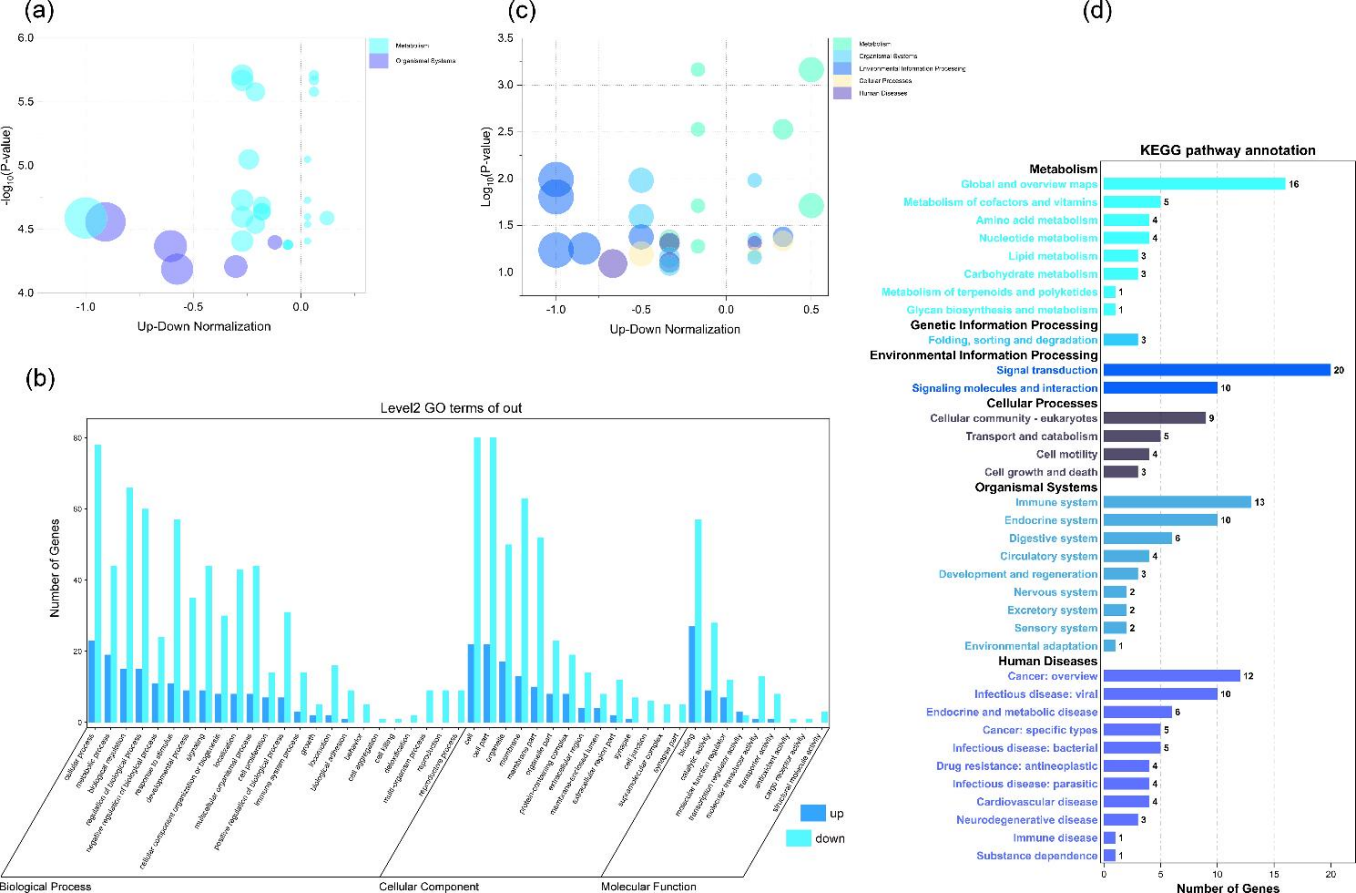
GO and KEGG enrichment analysis for mRNAs that were influenced by circRNAs-targeted-miRNAs: (**a**) Bubble chart of Top20 GO terms for targeted genes related to DE circRNAs; (**b**) Bar plot of GO enrichment terms in BP, CC, and MF categories for targeted genes related to DE circRNAs; (**c**) Bubble chart of Top20 KEGG Orthologys for targeted genes related to DE circRNAs; (d) Bar plot of KEGG enrichment pathways for targeted genes related to DE circRNAs

GO and KEGG (Kyoto Encyclopedia of Genes and Genomes) enrichment analysis were performed on mRNAs that were indirectly influenced by circRNAs. As shown in Fig. 6(a& b) and Table S6, the top20 GO terms that targeted genes enriched in were mostly associated with Cellular Component and Biological Process. Further refinement, the top20 GO terms were closely related to peptidyl-tyrosine phosphorylation, peptidyl-tyrosine modification, regulation of peptidyl-tyrosine phosphorylation, plasma membrane part, myelin sheath, intrinsic component of plasma membrane, external side of plasma membrane, integral component of plasma membrane, regulation of cytosolic calcium ion concentration, cellular calcium ion homeostasis, myelination, ensheathment of neurons, axon ensheathment, calcium ion homeostasis, regulation of biological quality, positive regulation of cytosolic calcium ion concentration, cellular divalent inorganic cation homeostasis, regulation of midbrain dopaminergic neuron differentiation, regulation of planar cell polarity pathway involved in axis elongation, negative regulation of planar cell polarity pathway involved in axis elongation and so on. And the KEGG enrichment analysis results shown by Fig. 6(c& d) and Table S7, top20 KEGG Orthologys distributed in 5 categories, included of Pyrimidine metabolism, Nicotinate and nicotinamide metabolism, Rap1 signaling pathway, Platelet activation, MAPK signaling pathway, Purine metabolism, Complement and coagulation cascades, Calcium signaling pathway, Thyroid hormone signaling pathway, Focal adhesion, Glycine, serine and threonine metabolism, Toxoplasmosis, Vitamin B6 metabolism, Ras signaling pathway, PI3K-Akt signaling pathway, Regulation of actin cytoskeleton, Vascular smooth muscle contraction, Central carbon metabolism in cancer, Human cytomegalovirus infection and so on.

### Enrichment analysis of sourcegene of circRNAs

**Fig. 7:**
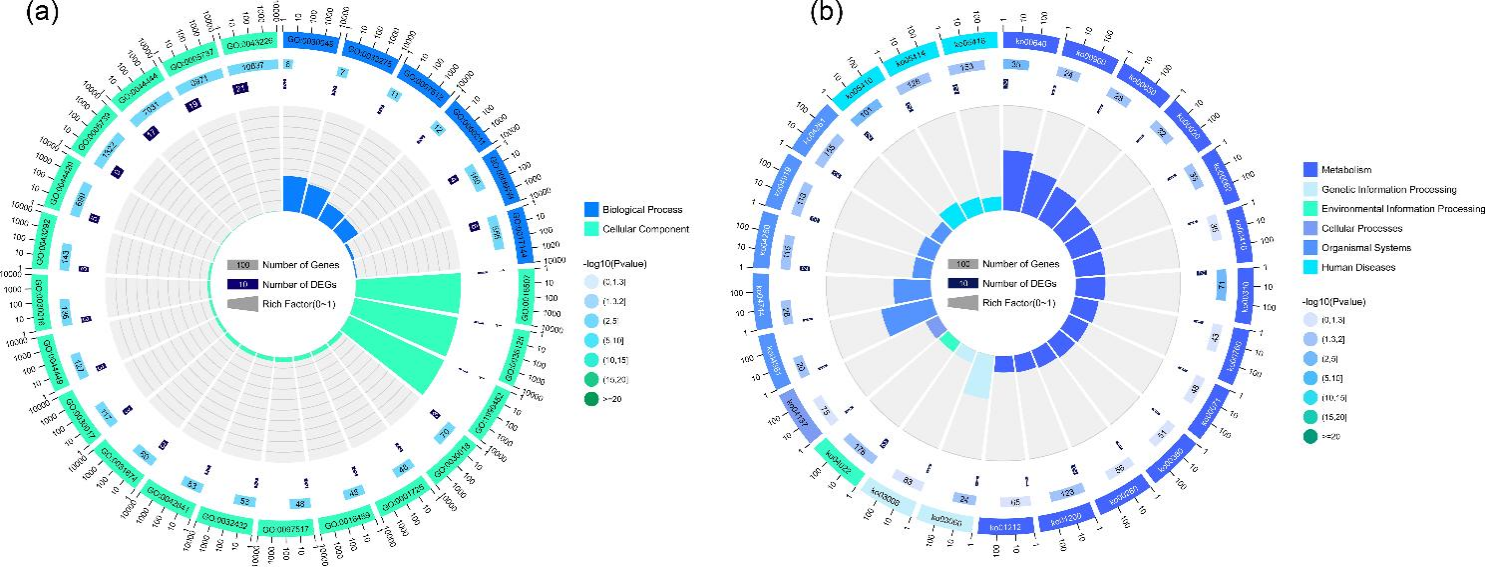
GO and KEGG enrichment analysis for sourcegenes of DE circRNAs: (**a**) Top25 GO terms function enrichment for sourcegenes of DE circRNAs; (**b**) Top25 KEGG Orthologys function enrichment for sourcegenes of DE circRNAs

CircRNA is usually produced by exons or introns of their sourcegenes, and the cyclization of circRNA probably affects the expression of the sourcegene [21]. We analyzed sourcegenes of 29 DE circRNAs from LQC and LFC, then KEGG and GO enrichment analysis were performed on them (Fig. 7). The result of top 25 GO terms in Fig. 7(a) illustrate that most terms were enriched in Cellular Component and 6 terms were enriched in Biological Progress. The information of all terms that were enriched have been stored in Table S8, and it is worth noting that most of the enriched genes were associated with the function of myofibers, such as sarcomere, contractile fiber part, myofibril, contractile fiber, stress fiber, myosin complex, contractile actin filament bundle, actin filament bundle, actomyosin, muscle filament sliding, actin-myosin filament sliding and so on. Figure 7(b) and Table S9 shown that top 25 KEGG Orthology of related genes, among them, there were 1, 1, 2, 3, 5 and 13 KEGG Orthology were classified to Cellular Processes, Environmental Information Processing, Genetic Information Processing, Human Diseases, Organismal Systems and Metabolism, respectively.

In order to verify the reliability of the RNA sequencing results, 3 circRNAs, 3 miRNAs and 8 mRNAs that differentially expressed in LQC and LFC were randomly selected and analyzed by RT-qPCR. The LQC served as a control group, RNA-seq and RT-qPCR results were consistent in the expression levels of each selected genes (Figure S1). Information about the primers used to amplify the selected genes has been placed in the Table S10.

## Discussion

### Differences in meat quality between longissimus dorsi from LQC and LFC may be related to the differences in myofiber composition

LFC and LQC are native Chinese cattle breeds, which have adapted to the hot and humid climate of southern China after long evolution; therefore, it is important to enhance their meat value potential. Most of the indicators used for meat quality assessment, such as pH decline, meat color, and tenderness, are related to the structure and metabolism of the muscle fibers [22, 23]. The rate of pH decline is often related to the amount of glycogen reserves in muscle tissue before slaughter and the content of mitochondria in different muscle fibers. When the glycogen level in muscle tissue is low, the rate of glycolysis decreases; therefore, the pH declines gradually because of the slow accumulation of lactic acid [24]. Muscles with more oxidative properties had a higher pH than those with more glycolytic properties after the animals were slaughtered [25], and the meat with a higher percentage of fast-twitch glycolytic fibers has a higher rate and degree of pH decline, it is commonly known in the field of red muscle. Furthermore, the rate of glycolysis and rate of pH decrease can affect changes of some proteins in muscle fibers at the post-mortem, such as tropomyosin, actin, troponin, and some enzymes on glycolysis, and these protein changes will be directly reflected in meat tenderness, flavor substances, color, and other measures of meat quality [8]. Meat color is an important point in determining meat quality, and it is a marketability criterion of meat because consumers often use this criterion to select and buy meat. Previous studies [26, 27] have shown that meat with low glycogen reserves tends to have higher ultimate pH, which leads to low light scattering and high oxygen consumption in meat surface, resulting in low lightness of the meat. The hue and chromaticity of meat color are primarily dominated by myoglobin (Mb) because the changes in the biochemical state of Mb cause the changes in meat color, particularly the degree of oxidation and reduction of Mb. In addition, Mb in fresh meat is present in four states, namely, deoxymyoglobin, oxymyoglobin (OxyMb), carboxymyoglobin (COMb), and metmyoglobin, in which OxyMb and COMb provide the meat with a bright cherry red color that is typical of fresh meat [28]. However, the four redox states of Mb are not constant, and many meat-endogenous factors can affect the color of meat by influencing the state of Mb, of which pH, muscle source, presence of antioxidants, lipid oxidation, and mitochondrial activity and the most prominent [29]. Beef tenderness has received considerable attention because it affects the return decision and satisfaction of consumers. The characteristics of the muscle fibers largely affect the tenderness of the meat, although the tenderness and texture of beef are also affected by the connective tissue and intermuscular fat in the muscle [30]. The activity of some protein hydrolytic enzymes changes with the decrease of the rate of pH, which leads to differences in glycogen metabolism by muscle fibers that indirectly affect meat tenderness [31, 32]. In addition, the number of muscle fibers affect the tenderness of meat. In general, tender meat has more muscle fibers per unit area and a smaller diameter [33]. In the present study, the pH of the longissimus dorsi of LQC decreased faster than that of LFC, which may be one of the reasons for the high lightness of the longissimus dorsi of LQC. With regard to redness, the longissimus dorsi of LFC has a higher level of redness than that of LQC, which may be due to some factors that keep the Mb more in the state of OxyMb or COMb in LFC. By analyzing the quantitative characteristics of myofibers per unit area, we found that the number of myofibers in LQC was less than that in LFC, and the diameter of myofibers was smaller. Such quantity characteristics of myofibers in LQC also confer a smaller shear force in LQC.

### ceRNA network points to the regulation of PI3K-Akt, MAPK, and calcium pathways

Considering that muscle fibers profoundly influence meat quality, the regulatory mechanism of myogenesis has been discussed comprehensively and extensively. Myogenesis is a physiological process that is influenced by various internal or external factors starting from fetal life. Among the factors, circRNA as a ncRNA has received considerable attention for its wide range of regulatory functions. circRNAs are dynamically expressed and abundant in muscle tissue of many species, including humans, cattle, goat, pig, chicken, and mice [9]. Although the functions of circRNAs remain largely unexplored, they can serve as miRNA sponges and further contribute to mRNA stability or protein production [34]. For example, circPTPN4 can sponge miR-499-3p, which regulates NAMPT expression, thereby promoting myoblast proliferation and differentiation and activating the fast-twitch muscle phenotype [35]. The overexpression of circCPE counteracts the inhibitory effect of miR-138 on cell proliferation and the accelerating effect on differentiation and apoptosis, and circRNAs can reduce the inhibitory effect of miR-138 on FOSC1, which is involved in myogenesis [36]. With the continuous improvement of sequencing technology and database, the construction of a ceRNA network has become an important tool for predicting circRNA function. Therefore, we performed Pearson correlation analysis to assess the association between DE circRNA and DE mRNA that exist between LQC and LFC, and enrichment analysis was performed for target mRNAs. Based on the results of KEGG analysis, we found that target mRNAs were primarily related to PI3K-Akt, MAPK, and calcium signaling pathways. PI3K-Akt has been widely proven to be associated with muscle hypertrophy and atrophy [37–40]. Several recent studies have also demonstrated the ability of circRNA to regulate the PI3K/AKT pathway, for example, the newly identified circRILPL1 as miR-145 sponge promotes myogenic cell growth by regulating the expression of the IGF1R gene to reduce the inhibitory effect of miR-145 on the PI3K/AKT signaling pathway [41]. The proliferation and differentiation of bovine myoblasts can be inhibited by circMEF2D, and circMEF2D can regulate the PI3K-AKT signaling pathway by competitively binding miR-486 [42]. Previous studies suggest that MAPK may be associated with myoblast cell cycle arrest, which is critical for initiating muscle differentiation in myogenic cells [43]. In addition, MAPK is a signaling pathway that drives the metabolic adaptation of skeletal muscle to exercise [44]. Numerous studies have shown that MAPK increases insulin-dependent glucose uptake and oxidative metabolism as well as mitochondrial oxidative phosphorylation in muscle during exercise [44–47]. In muscle tissue, intracellular stores of Ca^2+^, upon release, trigger the formation of actin crossbeams and the generation of contractile force [48]. Myocytes can sense and respond to changes in workload and activation patterns by regulating the gene expression and cellular metabolism of calcium signaling pathways [49, 50]. Stored calcium influx has become a mechanism by which the calcium signaling pathway is activated in response to the changing demands of myocytes [48, 51]. Most of the target mRNAs identified by the ceRNA network using LQC and LFC longissimus dorsi were enriched in pathways related to muscle metabolism and myogenesis. Therefore, these mRNA-related circRNAs and miRNAs might be effective tools for regulating muscle production and development.

### Cyclization of btacirc_00497 and btacirc_034497 may affect myogenesis

Apart from being a sponge for miRNA, circRNA can compete with linear splicing to play a functional role in gene regulation [15]. In this study, we attempted to use GO and KEGG enrichment analyses to organize and predict the function of genes that were affected by and were the source of DE circRNA. Based on the results of enrichment analysis, MYH6, MYH7, and nebulin (NEB) have high frequency in multiple pathways and GO terms that may associate with myogenesis and meat quality. Myosin is a motor protein that plays an important role in the contraction of animal skeletal muscle. Myosin consists of six subunits, two of which are myosin heavy chain (MyHC) subunits. MyHC isoforms play an irreplaceable role in muscle contraction because they have ATPase activity, which provides energy for muscle contraction [22]. In mammals, 11 known genes can encode MyHC, and these genes are highly conserved during evolution [52]. The four MyHC isoforms, namely, I, IIa, IIx, and IIb, are classified by different coding genes, which leads to four myofiber types. MyHC I is expressed in type I fibers, and IIa, IIx, and IIb are expressed in IIA, IIX, and IIB fiber types, respectively [53, 54]. The two subunits of the MyHC I heavy chain are composed of β-cardiac and α-cardiac encoded by MYH7 and MYH6, respectively [55]. Theoretically, the high expression level of MyHC I indicates that muscle fiber metabolism will have less efficient glycolysis, resulting in less lactate accumulation [56]. Low levels of lactic acid in the muscle tissue of live cattle can lead to a gradual decrease in beef pH rate postmortem [24]. Insufficient pH reduction in beef impairs meat color, tenderness, and shelf life [57]. Many studies have shown that feeding high-energy diet before slaughter is an effective way to increase muscle glycogen content and improve muscle pH reduction after slaughter, and this strategy may not become dependent on muscle fibers that have high MyHC I expression level [58, 59]. In our study, *btacirc_00497* in LFC longissimus dorsi has a high expression level, which is sourced from MYH6 and MYH7. However, our findings on the meat quality of LQC and LFC do not support our conjecture, that is, circRNA reduces β-MHC and α-MHC expression by competing for linear shearing of the transcripts of MYH6 and MYH7 and further influences the metabolic process of MyHC. We hypothesize that the expression level of MYH6 and MYH7 transcripts of LFC may be higher than that of LQC, so the probability of cyclization to *btacirc_00497* is also increased. Thus, although the factors affecting the cyclization of *btacirc_00497* are unclear, this gene may still serve as a potential lynchpin for the regulation of muscle fiber types. In addition, we identified *btacirc_034497*, which shares the same origin gene as NEB. Actin is present and functionally important in most eukaryotic cells, and the control of actin filament organization and structure is critical for many cellular functions. NEB stabilizes actin filaments in thin filament architecture, thereby regulating the filament length [60, 61]. Moreover, NEB regulates skeletal muscle contraction in skeletal muscle, and muscles from NEB knockout mice produce significantly less force than their wild-type siblings [62, 63]. Therefore, the factors affecting *btacirc_034497* cyclization have great application potential as important tools for NEB regulation.

## Materials and methods

### Sample collection

All experimental animals were sourced from the breeding farm (Meixian Country, Meizhou City, Guangdong province). Four Leiqiong cattle (LQC) and four Lufeng cattle (LFC) each, four-month-old, were humanely slaughtered, and the cattle of the same breed are similar in weight and body condition and raised in the same farm environment. Then longissimus dorsi tissues from 8 cattle were collected and snap-frozen in liquid nitrogen immediately to extract total RNA. Another portion of the longissimus dorsi tissue was trimmed into 0.5×0.5×1.0 cm pieces and immediately fixed in 4% paraformaldehyde for observation in tissue sections. In addition, any anesthesia or euthanizing agent was not used in our study.

### Analysis of muscle quality properties

After 24 hours of slaughter, pH was determined using Meat pH Meter HI99163 (HANNA, Italy) on the longissimus dorsi. The colorimetric parameters of the muscles were calculated using the L* a * b * system with a colorimeter OPTO-STAR Meat Color Tester (Matthaus, Germany) from the average of three random readings of each sample. And the evaluation of steaming losses was performed according to the project of Honikel [64]. During the shear force measurement, three samples of the longissimus dorsi after 72 hours of aging of each cattle (1cm×1cm×3cm) were measured three times perpendicular to the fiber direction. The final shear force was the average of the three readings, and the measurements were expressed in Pascal(Pa) [65].

### Muscle fiber characteristics

The tissues were after fixing by 4% paraformaldehyde for 24h, and then immersed in xylene alcohol (1:1, v/v), infiltrated and embedded in paraffin. Cross sections of 3µm thickness were prepared, stained with hematoxylin and eosin, observed under a microscope, and photographed (400×magnification). The number of muscle fibers and total cross-sectional area were subsequently evaluated with Image-Pro Plus V6.0 (Media Cybernetics Inc., Rockville, MD, USA).

### Preparation and sequencing for RNA-seq

TRIzol (Thermo Fisher, Shanghai, China) used to extract total RNA from the tissue samples. RNA quantity and purity were determined using Agilent 2100 Bioanalyzer and the RNA 6000 Nano Labchip Kit (Agilent, Santa Clara, USA). Epicentre Ribo-Zero™rRNA Removal Kit (Epicentre, America) were used to remove rRNA before the RNA-seq library construction. Then, the rRNA-depleted RNAs were then fragmented and reverse-transcribed to obtain cDNA libraries using the TruseqTM RNA sample prep Kit (Illumina, America). Small RNA-seq library construction was performed according to the instructions of NEBNext Multiplex Small RNA library Prep (Illumina, America), which was followed by TRIzol extraction. Then, the library was amplified and enriched using PCR technique, proprietary indexed adapters were then ligated to 5ʹ- and 3ʹ-termini, and later electrophoresed using 15% concentration of agarose gel to obtain the target fragments. Finally, all sequenced libraries were sent to the sequencing company (Personalbio, Shanghai, China) and sequenced by Illumina Hiseq4000 platform for paired-end sequencing after quality control.

### Bioinformatics Primary Analysis

The reference genome used in this experiment was Bos taurus ftp:(ftp.ensembl.org/pub/release-101/fasta/bos_taurus/dna). For miRNA, Hiseq Single-End mode sequencing data were mapped between reference genome sequences after quality control using miRDeep2 [66] software. The mature miRNA and precursor sequences of the species miRNAs were downloaded from miRbase (https://www.mirbase.org/). Then the de-duplicated sequences were mapped and annotate. Sequences that were not annotated with any information were analyzed using mireap (http://sourceforge.net/projects/mireap). DESeq V1.18.0 [67] was performed to analyze miRNAs for differential expression and filtered by |log2FoldChange|>1 and *P-value* < 0.05 to filter out differentially conserved miRNAs.

For the circRNA and mRNA, downstream data were used for initial evaluation of row data using FastQC v0.11.9 software after RNA-seq was completed. Cutadapt v2.6 was used to filter lower quality data as well as adepters. The obtained clean data were used for the subsequent analysis of circRNA and mRNA. During the analysis of mRNA, the upgraded HISAT2 (http://ccb.jhu.edu/software/hisat2/index.shtml) software of TopHat2 was used to map reads with reference genes. The differentially expressed transcripts were analyzed using DEseq, and the differentially expressed genes were screened for |log2FoldChange| > 1, *P-value* < 0.05. During the circRNA prediction analysis, the 20 bp of two ends of the reads that were not matched on the HISAT2 were realignment and used as Anchors. Ffind_circ [68] was used for circRNA identification. CircRNA expression was analyzed for differential expression, and the screened standard was |log2FoldChange| > 1 and *P-value* < 0.05. GO (Gene Ontology) and KEGG (Kyoto Encyclopedia of Genes and Genomes) enrichment analysis [69–71] were performed using DAVID (https://david.ncifcrf.gov/). In addition, miRNA targeting relationships to circRNA and mRNA were predicted using miRanda and psRobot software.

### RT-qPCR validation

After obtained sequencing results, RNA was extracted from the longissimus dorsi tissue using TRIzol (Thermo Fisher, Shanghai, China). RNA was reverse-transcribed to cDNA using the method provided in the introductions of PrimeScript RT Reagent Kit-Prefect Real Time (Takara, Beijing, China) for validation of mRNA and circRNA. miRNA 1st Strand cDNA Synthesis Kit (by stem-loop) (Vazyme, Nanjing, China) was used for miRNA reverse transcription. 2×Ultra SYBR Green qPCR Mix (CISTRO, Shanghai, China) was used for RT-qPCR in an Applied Biosystems QuantStudio 5 (Thermo Fisher, Shanghai, China). The reaction mixture containing, reaction conditions and primer sequences for the RT-qPCR validation procedure are stored in Table S10. The results were statistically analyzed using the 2^-ΔΔCT^ method [72]. All data are expressed as the average of 4 independent experiments.

### Statistical analysis

The comparative analysis of two groups was performed using unpaired independent t-test. SPSS 25.0 (SPSS Inc., Chicago, IL) and GraphPad prism 9 was used for statistical analyses. Results were considered statistically significant differences at *P* < 0.05 and were expressed as the mean ± Standard Deviation unless otherwise stated.

## Conclusions

In this study, we found that the pH drop and brightness of beef from LQC were significantly higher than those from LFC. In addition, the small cross-sectional area and diameter of muscle fibers produced better tenderness for beef from LQC. However, LFC was found to have superior redness compared with LQC. Furthermore, we identified several circRNAs related to muscle production and metabolism by ceRNA network building and analysis. By analyzing the source genes of DE circRNAs, we found that *btacirc_00497* and *btacirc_034497* may regulate the expression of MYH6, MYH7, and NEB by competing for linear shear, thereby altering the muscle fiber structure. Therefore, the ceRNA network-related genes and circRNA-derived genes identified in this study could be used as tools for regulating muscle production and meat quality of cattle grown in a subtropical environment.

### Electronic supplementary material

Below is the link to the electronic supplementary material.

Supplementary Material 1 Figure S1. Test of molecular biological validation about RNA sequencing

Supplementary Material 2 Table S1. RNA sequencing information statistics

Supplementary Material 3 Table S2. Differentially Expressed Gene Catalog

Supplementary Material 4 Table S3. Differential expressed mRNA enrichment analysis

Supplementary Material 5 Table S4. circRNA information from find_circ

Supplementary Material 6 Table S5. Co-expression Network Relationships and Network Analysis

Supplementary Material 7 Table S6. Target mRNA GO Enrichment Analysis

Supplementary Material 8 Table S7. Target mRNA KEGG enrichment analysis

Supplementary Material 9 Table S9. circRNA source gene KEGG enrichment analysis

Supplementary Material 10 Table S10. Primer and reaction system information

Supplementary Material 11 Table S8. circRNA source gene GO enrichment analysis

## Data Availability

The raw sequence data reported in this paper have been deposited in the Genome Sequence Archive (Genomics, Proteomics & Bioinformatics 2021) in National Genomics Data Center (Nucleic Acids Res 2022), China National Center for Bioinformation / Beijing Institute of Genomics, Chinese Academy of Sciences (**CRA007241** and **CRA007242**) that are publicly accessible at https://ngdc.cncb.ac.cn/gsa.
