# Supplementary material for: Expression profile and bioinformatics analysis of circRNA and its associated ceRNA networks in longissimus dorsi from Lufeng cattle and Leiqiong cattle
Source: BMC Genomics. 2023 Aug 29;24:499. doi: 10.1186/s12864-023-09566-0 (PMC10466722; doi:10.1186/s12864-023-09566-0)
Supplement: Supplementary file 1 — Supplementary Material 1 Figure S1. Test of molecular biological validation about RNA sequencing [file 12864_2023_9566_MOESM1_ESM.pdf]

Fig S1

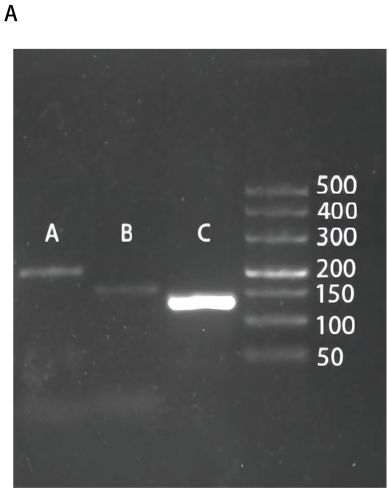
A
A
B
C
500
400
300
200
150
100
50

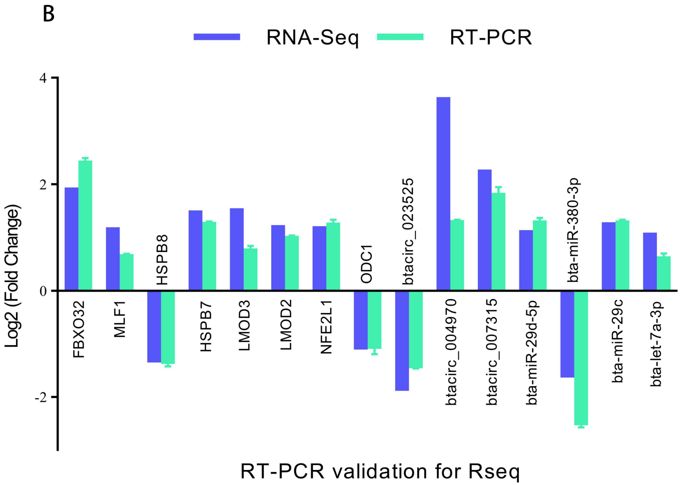
B
RNA-Seq
RT-PCR
Log2 (Fold Change)
4
2
0
-2
FBXO32
MLF1
HSPB8
HSPB7
LMOD3
LMOD2
NFE2L1
ODC1
btacirc_023525
btacirc_004970
btacirc_007315
bta-miR-29d-5p
bta-miR-380-3p
bta-miR-29c
bta-let-7a-3p
RT-PCR validation for Rseq

Fig.S1 Test of molecular biological validation about RNA sequencing.

(A) Agarose gel electrophoresis of primers for amplifying circRNAs;

(B) RT-PCR validation of RNA sequencing;
